# Gleanings

**Published:** 1897-10

**Authors:** 


### GLEANINGS.

THE EFFECTS OF POTASSIUM IODIDE ON A LARGE TUMOR.—The published account by Malkomis on the cure of a schirrhous cord by large doses of iodine given internally induces me to publish the following: On March 5, 1896, a seven-year-old Wallachian, which had a large tumor on the abdomen, extending from the elbow to the flank, was brought to the surgical clinic of the Stuttgart High School. The surface of the swelling was covered with scales and cracks caused by repeated blisters. The consistence of the tumor was firm and harder than the muscles of the croup and back. From a clinical point of view the author deemed the growth a sarcoma.

On account of the great dimensions of the tumor, an operation was deemed inexpedient. The swelling was well rubbed with Lugol's ointment (iodine, 1 part; pot. iod., 10; lanolin and vaselin, of each 50), and then covered with a pressure-bandage, inside of which was placed a piece of cloth soaked in 2 per cent. lysol solution, and the whole covered with a folded saddle-blanket. At first the horse received very little drinking-water. Eserine was then given, also a ball containing aloes, calomel, and croton oil. On every third day thirty grains of potassium iodide was given in the drinking-water. Later the horse took daily twenty grains of potassium iodide in the drinking-water. The treatment proved wholly favorable, the tumor got small and softer, and four weeks after the horse was at work again.—*Veterinary Record.*

Now that contagion is known to be the principal factor in the spreading of bovine tuberculosis, the importance of separating healthy animals from those that are infected is generally recognized. Up to this time this has been a very difficult undertaking, as it was almost impossible to say with certainty which animals were affected and which not. It is possible for an animal to be a prolific source of infection, and yet show no physical signs of tuberculosis. To-day, by the use of tuberculin, all the diseased animals can be singled out, even if the disease is in its earliest stages and the parts affected very insignificant. Concentrated tuberculin is not adapted to administration, and proper dilution at the time of use is very difficult. For this reason tuberculin is now prepared diluted ready for injection, and is supplied in vials containing from 2 to 250 doses, according to the quantity desired.

